# 2226 Influence of alcohol use disorder and comorbid psychopathology on discounting of delayed rewards

**DOI:** 10.1017/cts.2018.169

**Published:** 2018-11-21

**Authors:** Julia Swan, Joshua L. Gowin, Matthew E. Sloan, Reza Momenan, Vijay A. Ramchandani

**Affiliations:** National Institutes of Health

## Abstract

OBJECTIVES/SPECIFIC AIMS: Alcohol use disorder (AUD) has been associated with greater discounting of delayed rewards relative to healthy controls. The relationship, however, has been inconsistent, likely because previous studies had relatively small sample sizes and inadequately controlled for comorbid psychopathology and substance use. In the present study, we analyzed one of the largest clinical research samples to date to assess the influence of alcohol use on delay discounting, and examine the influence of confounding variables including substance use disorder. METHODS/STUDY POPULATION: In total, 801 participants completed a delay discounting task where they chose between smaller, immediately available monetary amounts ($0–$90) and $100 available after a delay of 7–30 days. Delay discounting behavior was summarized as the natural log of *k*, a constant derived from a hyperbolic discounting equation. Participants also completed Structured Clinical Interviews for DSM-IV disorders, 90-day Timeline Followback interviews, and the Fagerström Test for Nicotine Dependence. Participants were divided into 4 groups: healthy controls (n=298), past AUD (n=69), and current AUD with (n=224) and without (n=210) comorbid psychopathology or substance use disorder. Kruskal-Wallis test was used to examine the effect of group on delay discounting. RESULTS/ANTICIPATED RESULTS: There were significant differences in the distribution of delay discounting scores by group (*H*=80.195, *p*<0.001). Healthy controls and past AUD showed lower levels of delay discounting than current AUD and current AUD+comorbidity groups with medium effect sizes (Cohen’s *d*=−0.635 and Cohen’s *d*=−0.614, respectively). There were nearly no differences between current AUD with and without comorbid psychopathology groups (Cohen’s *d*=−0.024). The past AUD group showed almost no difference relative to the healthy control group (Cohen’s *d*=0.007). DISCUSSION/SIGNIFICANCE OF IMPACT: Individuals with current AUD were shown to discount rewards greater than those without current AUD, although comorbid psychopathology did not significantly affect discounting. Surprisingly, individuals with past AUD were more similar to controls than to those with current AUD. Our findings suggest that current problematic alcohol use is related to greater discounting of delayed rewards, but comorbid diagnoses do not significantly impact this relationship. However, once problematic patterns of alcohol use cease, delay discounting appears to return to levels comparable to healthy controls.

